# Positive effects of a perioperative training intervention in Ivor Lewis oesophageal surgery: a randomised, controlled multicentre trial

**DOI:** 10.1186/s12893-025-03416-4

**Published:** 2025-12-11

**Authors:** Monika Fagevik Olsén, Sara Löthgren, Ulrika Trölle, Elina Elf, Sophie Toomväli-Petersson, Michael Hermansson, Eva Hammerlid, Ulrika Smedh

**Affiliations:** 1 Department of Health and Rehabilitation/Physiotherapy, Institute of Neuroscience and Physiology, Sahlgrenska Academy, Gothenburg University, Box 100, Gothenburg, SE 405 30 Sweden; 2 Department of Surgery, Institute of Clinical Sciences, Sahlgrenska Academy, Gothenburg University, Gothenburg, Sweden; 3 Department of Surgery, Sahlgrenska University Hospital, Gothenburg, Sweden; 4 Department of Physiotherapy, Sahlgrenska University Hospital, Gothenburg, Sweden; 5 Department of Physiotherapy, Örebro University hospital, Örebro, Sweden; 6 Skånes University Hospital, Lund, Sweden; 7 Karolinska University Hospital, Stockholm, Sweden; 8 Linköping University Hospital, Linköping, Sweden; 9 Department of Otorhinolaryngology-Head and Neck Surgery, Sahlgrenska University Hospital, Institute of Clinical Sciences, Sahlgrenska Academy, University of Gothenburg, Gothenburg, Sweden

**Keywords:** Oesophageal surgery, Physical activity, Respiratory muscle training

## Abstract

**Background:**

The effects of peri-operative training interventions in connection with oesophageal surgery have not been thoroughly investigated. The aim of this randomised, controlled, single-blind study was to evaluate a peri-operative physical training programme in patients undergoing oesophageal cancer resection surgery due to cancer of the oesophagus or the gastro-oesophageal junction.

**Methods:**

One hundred patients scheduled to undergo Ivor Lewis oesophagectomy in one of five university hospitals in Sweden were randomised to a control group or intervention including respiratory muscle training, strength training, and increased physical activity before surgery and up to 3 months postoperatively. Outcome measures were physical capacity, respiratory muscle strength, spirometry, grip strength, and chest mobility. Questionnaires regarding physical activity and function, recovery, and health-related quality of life were completed at inclusion and 3 and 12 months postoperatively.

**Results:**

We did not find significant differences between the groups in physical capacity during follow-up. However, the intervention group performed significantly better than the control group in maximal inspiratory pressure (Δ18%), maximal expiratory pressure (Δ18%), and peak expiratory flow (Δ12%) 3 months postoperatively (*p* < 0.05). Patients in the intervention group also developed fewer pulmonary complications (*p* = 0.019). We did not find differences between the groups in recovery or health-related quality of life.

**Conclusion:**

A peri-operative training intervention including respiratory muscle training had a positive impact on respiratory function and pulmonary complications. However, neither strength training nor increased physical activity had any effects. Thus, respiratory muscle training may be offered as a peri-operative regimen in oesophageal cancer surgery.

**Trial registration:**

FoU i VGR 238,651 (Released Dec 15, 2017), Clinical Trials NCT03452319 (Released Feb 18, 2018).

**Supplementary Information:**

The online version contains supplementary material available at 10.1186/s12893-025-03416-4.

## Introduction

Oesophageal resection for cancer of the oesophagus or cancer of the gastro-oesophageal junction is a major surgical procedure with a high risk of comorbidities and prolonged catabolic effects [1]. Patients with potentially curable oesophageal cancer often exhibit reduced physical performance at diagnosis due to various factors, including weight loss from cancer-induced dysphagia, leading to decreased nutrient intake and fatigue [2]. The patient group typically consists of males, often with a history of smoking, who were frequently overweight before symptom onset [3–5]. Due to the extent of the surgery and the risks of surgical and cardiorespiratory complications, not all patients are eligible for surgery because of severely reduced physical performance levels and/or serious comorbidities. The high risk of lung complications is also associated with the thoracic part of the procedure and the overall extent of the surgery [6]. Given the progressive weight loss these patients often experience, a well-recognised clinical focus during the work-up period is ensuring that nutritional needs are met to counteract catabolism and reduce peri-operative risks. The potential value of additional physical training before and after the surgical procedure is less established.

In recent years, several studies have explored the value of physical training as part of treatment programmes to improve outcomes. A review by Bolger et al. [7] identified 12 studies, including 5 randomised controlled trials. These interventions featured various pre-operative exercise programmes, with some incorporating inspiratory muscle training (IMT). The authors concluded that prehabilitation may be beneficial, but larger randomised controlled trials are necessary to fully assess its effects.

The review also examined postoperative interventions [7], concluding that postoperative rehabilitation was linked to improved clinical outcomes. In a previous randomised controlled study by our research group, we found that patients who participated in a postoperative physical training programme benefitted from the structured regimen [8]. Although patients did not demonstrate increased functioning or strength compared to their individual pre-operative levels, they recovered their pre-operative physical functioning significantly better than the control group. Similar effects have been observed by others [9].

Although benefits of specific training, such as IMT, have been shown during recovery after Ivor Lewis oesophageal resection, few scientific studies and no clinical guidelines support a targeted training programme before oesophageal surgery. In a randomised pilot study involving patients with locally advanced oesophageal cancer, adding physical training during oncological treatment appeared to yield some improvements, but the study was small [10]. Our previous positive findings [8], combined with the lack of solid scientific evidence for prehabilitation training in this patient group [7], led us to hypothesise that introducing a training programme after neoadjuvant treatment but before oesophageal resection surgery, along with postoperative training, would enhance physical performance, respiratory function, thoracic mobility, and quality of life.

The aim of this randomised, controlled, single-blind study was to evaluate a peri-operative physical training programme in oesophageal cancer resection surgery due to cancer of the oesophagus or of the gastro-oesophageal junction (Siewert I-II).

## Materials and methods

Between January 2018 and May 2022, a total of 100 patients who underwent oesophageal resection surgery at Sahlgrenska University Hospital Gothenburg, Karolinska University Hospital Stockholm, Örebro University Hospital, Skåne University Hospital Lund, or Linköping University Hospital were recruited into this study. All patients diagnosed with oesophageal cancer who planned to undergo oesophageal resection were eligible for inclusion. Patients were excluded if they planned to undergo surgery within 2 weeks after inclusion, had difficulties understanding spoken and written Swedish, or if they had another injury or disease that restricted thoracic mobility or the possibility of performing the intervention, such as rheumatoid arthritis or spinal injury. The study protocol was approved by the Swedish Ethical Review Authority (Registration number: 542 − 17).

Eligible patients at each hospital were given oral and written information about the study during a standard visit to the surgical outpatient clinic after decisions had been made about therapy. The patients who accepted to participate, gave their written consent, and were scheduled for surgery were contacted directly by the physiotherapist in charge to plan an inclusion visit. Those who underwent radio-chemotherapy before the operation were contacted after the neo-adjuvant treatment.

At the end of the inclusion visit by the physiotherapist, the patients were randomised to the intervention or control group (1:1) using an electronic random number table. Randomisation was stratified according to hospital and sex. The randomisation process was performed by a person who was not involved in the study.

Twenty-nine patients were lost to follow-up 3 months after surgery for the following reasons: surgery was cancelled, a different operation was performed, surgery was moved to an earlier date (not allowing for the training period), the patient declined participation in the follow-up (mainly because of COVID-19 restrictions), patient was deceased, or logistical reasons. The demographic data of the patients who participated in any of the postoperative follow-ups and those who were lost to follow-up are given in Table 1. There were no differences between the patients who were randomised to the intervention or control group or between the patients who were lost to follow-up compared to those who participated in the 3-month follow-up.

**Table 1:** Demographic and perioperative data of the patients who participated in the trial or were lost to follow-up. Mean (sd), n (%)

		Intervention group *n* = 52	Control group *n* = 48	*p*-value	Lost to follow-up *n* = 25
Age, years		67.4 (8.2)	67.1 (7.1)	0.294	66.5 (7.1)
Sex, male/female		42/10	38/10	0.841	20/5
BMI, kg/m^2^		25.5 (3.9)	25.0 (3.6)	0.529	24.4 (3.8)
Weight loss since symptoms, %		-6.0 (6.2)	-8.3 (10.1)	0.100	-8.1 (10.0)
Smokers Yes/X-smoker		8/14	6/13	0.903	3/4
FVC, % pred		97.1 (16.6)	97.2 (16.7)	0.789	97.9 (16.9)
FEV1, % pred		93.5 (17.5)	90.9 (15.2)	0.210	89.5 (14.0)
PEF, % pred		98.5 (17.5)	92.1 (17.6)	0.577	88.2 (17.5)
MIP, % pred		104.1 (28.3)	92.9 (25.4)	0.487	91.6 (28.4)
MEP, % pred		107.5 (29.0)	103.1 (31.6)	0.711	109.0 (18.3)
Neoadjuvant radio- & chemotherapy, n (%)		44	36	1.000	8
Physical function, 6MWD, % pred		96.1 (20.7)	91.1 (18.3)	0.687	84.7 (16.7)
WHO performance score	Asymptomatic	37	28	0.281	9
	Symptomatic, ambulant	11	9		5
	Symptomatic, < 50% in bed	0	2		
	Symptomatic, >50% in bed	0	0		
Neoadjuvant treatment		45	36	0.368	12
Type of cancer	Adenocarcinoma	28	24	0.528	10
	Squamous cell carcinoma	6	5		2
	Not defined preop	6	2		2
Blood loss, mL		252 (371)	420 (530)	0.136	
Duration of surgery, h		7.6 (2.0)	7.4 (1.7)	0.203	
Type of surgery, thorax	Thoracoscopic	10	10	0.895	
	Thoracotomy	37	29		
Type of surgery, abdomen	Laparoscopic	42	30	0.164	
	Laparotomy	7	9		
cTNM-classification	T 0/1/2/3/4	6/3/5/17/2	4/8/6/13/2	0.780	
	N 0/1/2/3	20/8/6/6	21/3/5/2	0.348	
	M 0/1	38/2	30/1	0.712	

*BMI* Body Mass Index, *FVC* Forced Vital Capacity, *FEV1* Forced Expiratory Volume during 1 s, *PEF* Peak Expiratory Flow, *MIP* Maximal Inspiratory Pressure, *MEP* Maximal Expiratory Pressure, *Pred* predicted, *Preop* Preoperative

### Intervention group

The purpose of the intervention was to increase daily physical activity and optimise respiration before surgery, as well as to increase the postoperative recovery. To increase the level of physical activity, the patients received information about the importance of a good physique before the procedure. The patients were told to be physically active at a level corresponding to moderate effort for 30 min daily. Patients who, even before inclusion, rated themselves at 5–6 on the activity scale according to Grimby [11] were asked to maintain this level until surgery. Appropriate activity was discussed with the physiotherapist who followed up on the conversation by phone after 1 week. In addition, the patients received a training programme with four strength training exercises to perform daily and could be included in the 30 min of extra training.

IMT was conducted according to van Aldrichem [12] with a modification; daily, patients performed three sets of 10 forceful and deep breaths from the residual volume, followed by a post-inspiratory pause. The resistance was set to 60% of the maximum inspiratory pressure (MIP) and increased if it became too easy. This training was combined with expiratory muscle training (EMT), targeting at least 50% of the maximum expiratory pressure (MEP). The goal of adding EMT was to strengthen the expiratory muscles, making it easier for patients to clear secretions postoperatively [13]. Patients performed three sets of 10 forceful exhalations from total lung capacity daily. A flow-dependent device (PEP/RMT set, Mediplast, Malmö, Sweden) was used, and patients were provided with a manometer to ensure the correct training pressure. Training was done using a mask or mouthpiece based on patient preference. All training was unsupervised and recorded by the patients in logbooks both pre-operatively and postoperatively.

Postoperatively, the patients were told to be physically active again as soon as possible by walking and performing other light activities. The goal was to be physically active for at least 30 min/day within 2 weeks after leaving the hospital. After discharge, the patients continued with IMT and EMT daily during the first month and three times per week thereafter until the 3-month postoperative visit.

### Control group

The patients in the control group were encouraged to continue their usual physical activity pre-operatively and postoperatively.

During the peri-operative period, the patients in both groups were mobilised according to the established hospital routine. In addition, they performed breathing exercises with PEP (Positive Expiratory Pressure) to increase lung volumes and evacuate secretions [14]. Upon discharge, patients received exercise programmes to increase mobility and strength in the chest and shoulders [8].

A follow-up at the hospital took place 3 months after surgery. The same tests were carried out and questionnaires answered as at inclusion. The tests were carried out by an individual blinded to the patient’s group allocation. Another follow-up was performed 1 year after the procedure, at which the patients only filled out the questionnaires.

### Outcomes

Several tests were performed at inclusion and 3 months postoperatively to measure the outcomes of the study.

### Physical capacity

The 6-minute walking distance (6MWD), where the patients walked a distance of 30 m (indoors, back and forth in a corridor) for 6 min, was measured according to international guidelines [15]. The distance covered during the time was noted in metres [15].

The timed-stands test was performed by asking the patient to sit down and stand up 10 times from a chair 45-cm-high without using the arms for support. Time to complete the task was recorded in seconds [16].

### Respiratory muscle strength

MIP/MEP were measured using a Micro RPM (Care Fusion, Yorba Linda, CA, US) while the patient was sitting with a nose clip [17]. The test was performed according to guidelines from the European Respiratory Society and at least three attempts made; the best value was noted. MIP was measured at the residual volume and MEP at the total lung capacity.

### Spirometry

Standardised spirometry was performed using an EasyOne spirometer (ndd Medical Technologies, Switzerland). The forced vital capacity (FVC), forced expiratory volume in one second (FEV1), and peak expiratory flow (PEF) were measured while the patient was sitting according to the instructions from the European Respiratory Society [18]. To avoid an impact of sex, age, and height, percent predicted in reference values were calculated according to the reference equations for spirometry.

### Grip strength

Maximal grip strength was measured by a Jamar handheld dynamometer (Sammons Preston, Bolingbrook, Illinois, US) in the sitting position with the forearm on an armrest [19]. The patient made three attempts, and the best result was noted.

### Physical function

The patients assessed their level of physical function using the Disability Rating Index (DRI) questionnaire [20]. The patients were asked to estimate their current function in 12 activities on visual analogue scales, from walking and doing household activities to intensive sport activities. Lower values indicated limited physical function.

### Physical activity level

The level of physical activity on a weekly basis was assessed by the Grimby scale [11]. The six levels on the scale ranged from mostly sedentary (level 1) to intensive physical training >3 h/week (level 6).

### Quality of recovery

The Postoperative Recovery Profile (PRP) was used to assess the quality of recovery [21]. It includes 19 items grouped into five dimensions: physical symptoms, physical function, psychological, social, and activity. The items are rated on a four-grade Likert scale from none to severe impact. Global recovery was calculated according to Allvin et al. [21].

### Health-related quality of life questionnaires

In this study, the European Organisation of Treatment and Research of Cancer (EORTC) quality of life questionnaires were used: Core 30 (EORTC QLQ-C30), fatigue (EORTC QLQ-FA12), and a diagnosis-specific questionnaire (EORTC QLQ-OG25). These were all developed according to the EORTC guidelines and have proved useful in both studies and clinically.

The EORTC QLQ-C30 was developed for all types of cancer and consists of five function scales and nine symptom/problem scales together with a global quality of life scale [22]. The EORTC QLQ-FA12 poses three scales for fatigue (physical fatigue, emotional fatigue, and cognitive fatigue) and two single items. Only the scales are shown in this study [23]. The EORTC QLQ-OG25 is a diagnosis-specific questionnaire for cancer of the oesophagus and the gastro-oesophageal junction and includes one functional scale for body image and 15 symptom scales (dysphagia, eating, reflux, odynophagia, pain and discomfort, anxiety, eating with others, dry mouth, trouble with taste, trouble swallowing saliva, choking when swallowing, trouble with coughing, trouble with talking, weight loss, and hair loss) [24].

Most of the questions were answered according to a four-point Likert scale and linearly transformed to a score ranging from 0 to 100. A high score for a functional scale and the global health scale represents a high level of functioning, but a high score for a symptom scale represents a high level of symptoms/problems [25]. A difference ≥ 10 points is considered a clinically significant difference between measurement points and between study groups [26].

### Postoperative surgical variables and complications

Data regarding surgical variables were extracted from the Swedish national registry for gastro-oesophageal cancer (NREV). The data included duration of surgery, blood loss, procedure details, Clavien-Dindo classification, and pulmonary and cardiovascular complications.

### Statistical analysis

The primary outcome, the 6MWD, was measured previously to be, in average, 400 m (standard deviation [SD] 100 m) 2 weeks after oesophageal surgery [27]. The minimal clinically important difference (MCID) in this patient group is missing, but the value has been defined as 32 m for heart failure [28] and 54 m for COPD [29]. As the oesophageal cancer patients often have cardiopulmonary co-morbidities, the difference between the groups in the present study was calculated to be at least 60 m. Therefore, the analysis was based on a walking distance of 400 m (SD 100), a difference between the groups of 60 m, power of 0.8, and alpha value of 0.05, which gave a group size of 44 individuals. To compensate for dropouts, 50 patients were planned to be recruited for each group. This group size was defined as sufficient to also detect the MCID in the second primary outcome, MIP [30].

The results of the study were analysed according to intention-to-treat and per protocol analyses. The results are presented as mean and SD, median with the minimum and maximum, and 95% confidence interval (CI) for the mean. Parametric statistics, t-test, ANOVA, and Fisher’s non-parametric permutation test were used for between-group and within-group analyses regarding continuous variables. The CI for the mean difference between groups was based on Fisher’s non-parametric permutation test. Non-parametric statistics, the Mann-Whitney U test, and the Kruskal-Wallis test were used for categorical variables. For dichotomous variables, chi-squared was used.

A difference ≥ 10 points in the items on the quality-of-life questionnaires was considered a clinically significant difference between measurements and between study groups as defined by Osoba et al. [26].

## Results

**Fig. 1:**
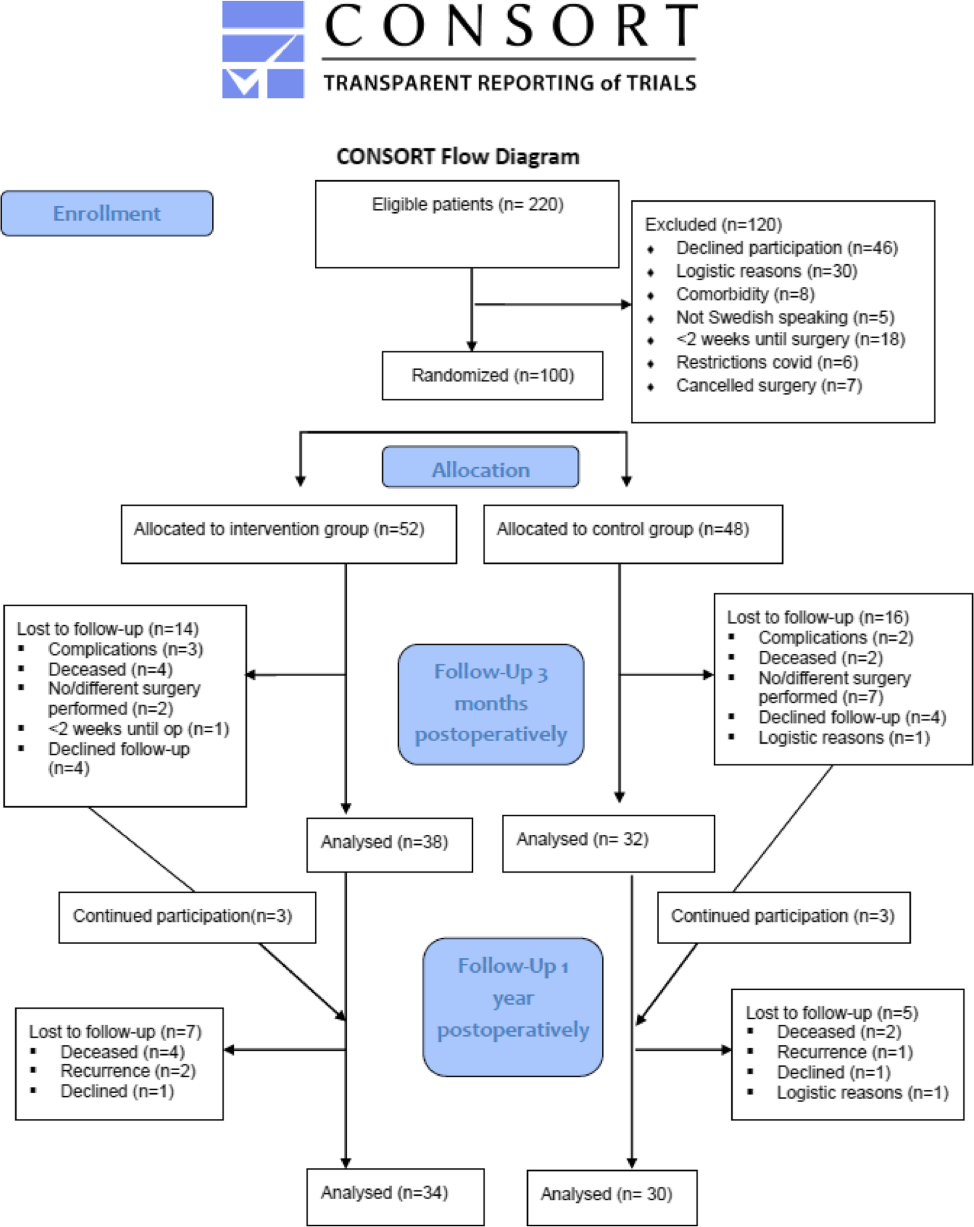
Flowchart of the trial

Of the 100 included patients, 71 participated in the follow-up 3 months after discharge (Fig. 1). Of those who participated, 7 declined the clinical visit (intervention group, *n* = 1; control group, *n* = 6) due to restrictions during the COVID-19 pandemic, but they completed the questionnaires. At the 1-year follow-up, the questionnaires were completed by 64 patients (intervention group, *n* = 34; control group, *n* = 30).

### Adherence to the intervention

The logbooks revealed that the patients in the intervention group pre-operatively performed the IMT and strength exercises 6–7 times per week. In addition, they were physically active > 220 min/week. Postoperatively, they performed the IMT 6–7 times per week in the first month and > 4 times per week thereafter. The strength training was performed > 4 times per week. The patients were physically active 76 min on average during the first week after discharge but increased the time to 179 min during the second week, with an average > 150 min/week during the whole study period.

### Postoperative complications

The number of complications is presented in Table 2. There were no significant differences between the groups in regard to the Clavien-Dindo classification or cardiovascular complications. One patient in the control group developed a pulmonary insufficiency and needed reintubation (vs. none in the intervention group, *p* = 0.461). In addition, 4 patients in the control group and 1 in the intervention group were diagnosed with pneumonia (*p* = 0.174). However, pulmonary complications overall were significantly more common in the control group (9 vs. 2, *p* = 0.019).

**Table 2:** Postoperative complications in the intervention and control group

		Intervention group *n* = 38	Control group *n* = 32	*p*-value
Pulmonary insufficiency needing reintubation, n		0	1	0.461
Pneumonia, n		1	4	0.174
Pulmonary complication*, n		2	9	0.019
Cardiovascular complication, n		1	1	0.910
Thromboembolism, n		0	1	0.461
Pulmonary embolism, n		0	2	0.209
Clavien Dindo	0	30	19	0.228
	I	2	1	
	Ii	6	5	
	IIIa	1	5	
	IIIb	2	2	
	IVa	0	2	
	IVb	0	1	

*Defined as at least one of following: pneumonia. pneumothorax, atelectasis, respiratory failure, acute respiratory distress syndrome, acute aspiration or tracheobronchial injury

### Three-month clinical follow-up

**Fig. 2:**
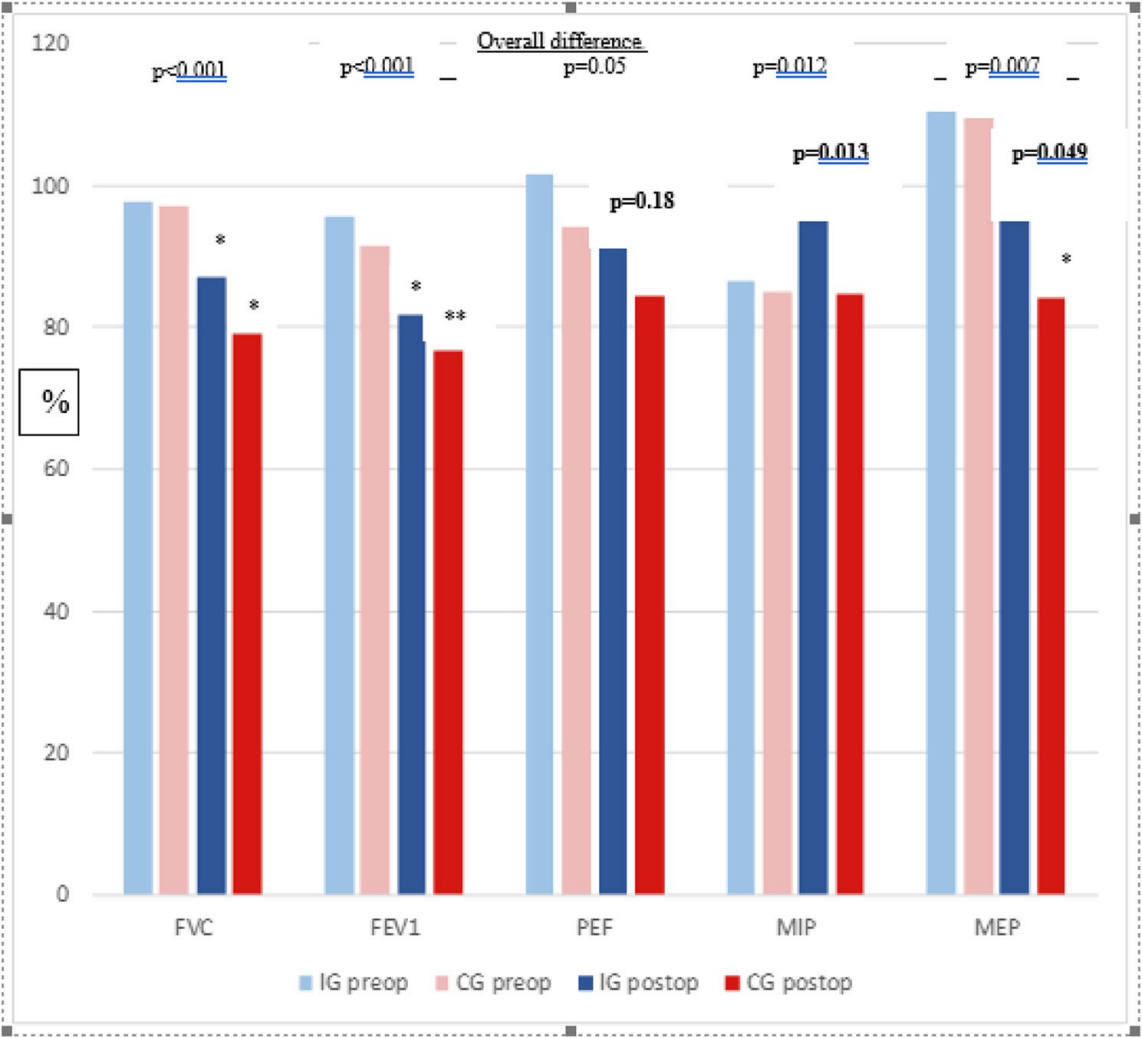
Spirometry and respiratory muscle strength after thoracoabdominal oesophageal surgery. IG: Intervention Group, CG: Control Group, * p<0.05 in difference to preoperative value, ** p<0.01 in the difference to preoperative value. Bold type: p-value of difference between intervention and control group postoperatively

The results of the tests performed at the clinical visit at 3 months are presented in Table 3, Supplementary Tables 1, and Fig. 2. The patients in both groups performed well during the 6MWD, with an average level > 90% of the predicted distance and no differences between the groups. The timed-stands test was more decisive, with 65% of the predicted time in the intervention group and 62% in the control group pre-operatively, and with a marginal difference postoperatively but no differences between the groups.

**Table 3:** Results, of predicted value (%), of the tests of physical function in those who participated in the clinical follow-up three months after Ivor Lewis esophagectomy. Mean (standard deviation)

	Inclusion			Change between inclusion and three months				
	Intervention group *n* = 52	Control group *n* = 48	*p*-value	Intervention group *n* = 35	Control group *n* = 26	*p*-value	difference between groups, Mean (95%CI)	Effect size
6MWD, % pred	96.0 (16.0)	94.3 (19.6)	0.718	-3.2 (17.4)	-0.7 (14.4)	0.56	-2.5 (-11.0; 5.6)	0,157
Timed stands test, % pred	65.1 (27.6)	61.6 (18.9)	0.577	-1.0 (14.0)	1.0 (16.0)	0.61	-2.00 (-9.6; 5.8)	0.134
Grip strength, % pred	115.5(45.3)	112.8(39.6)	0.810	-10.1 (27.4)	-15.6 (34.5)	0.48	5.4 (-10.4; 20.1)	0.178

*6MWD* 6 min Walk Distance

Regarding pulmonary variables (Supplementary Table 1), analyses between the groups showed that patients in the intervention group performed significantly better than the controls postoperatively (*p* < 0.05) in regard to MIP (Δ18%), MEP (Δ18%), and PEF (Δ12%). Pre-operatively, grip strength was higher than predicted in both groups (115% vs. 113%) and, despite a decline due to surgery, they performed the test within normal values.

In the within-group analyses of differences between the pre-operative and postoperative results, the patients in the intervention group had significantly lower spirometry (*p* < 0.01) and grip strength (*p* = 0.011) values 3 months after discharge compared to the values at inclusion. The patients in the control group also had significantly lower values in the same variables (*p* < 0.01), but also in MEP (*p* < 0.001).

There were no significant differences in the measured outcomes at 3 months between patients who underwent thoracotomy and those who underwent thoracoscopy (*p* < 0.05, data not shown).

### Questionnaires at the 3- and 12-month follow-ups

Regarding physical function measured by the DRI, there were no significant differences between the groups in the DRI score or disability level (Supplementary Table 1). The within-group analyses revealed that patients in both groups had a significantly lower DRI value (*p* < 0.05) 3 months after discharge compared to pre-operatively, but there was no significant difference after 12 months.

The level of physical activity and physical training decreased in both groups after surgery, but there were no differences between the groups (Supplementary Table 1). In addition, there were no significant changes within the groups over time (*p* < 0.05).

The level of recovery by the PRP is presented in Table 4. Three months after discharge, patients rated the items fatigue, appetite loss, sleeping, gastrointestinal function, muscle weakness, and re-establish everyday life on a level that corresponds to mild or moderate difficulties. Twelve months after discharge, the patients rated fatigue, sleeping difficulties, gastrointestinal function, muscle weakness, feeling down, and social activities highest, but they were all rated a median of “mild”. When combining the 19 items into the five dimensions, the items covering physical symptoms and physical functions were rated with less recovery than the psychological, social, and activity items. In total, six patients in each group (16% of intervention group, 19% of control group) rated that they were fully or almost fully recovered 3 months after discharge, and 11 (33%) and 16 (21%) patients, respectively, rated that they were fully recovered after 1 year, but the differences were not significant between the two groups. In contrast, one-third of the study cohort rated that they were not at all recovered after 3 months and 20% noted a lack of recovery after 12 months. No significant differences were found between the groups.

**Table 4:** Distribution of the patients´ reported level of recovery (by Allvin´s postoperative recovery Profile) three and twelve months after discharge from hospital after esophagectomy. Mean (standard deviation)

		3 months follow-up			12 months-follow-up		
		Intervention group *n* = 38	Control group *n* = 32	*p*-value	Intervention group *n* = 33	Control group *n* = 30	*p*-value
Global scale, level of recovery	Fully	1 (3%)	2 (6%)	0.813	3 (9%)	6 (20%)	0.360
	Almost fully	5 (14%)	4 (12%)		8 (24%)	10 (33%)	
	Partly	17 (46%)	11 (34%)		13 (39%)	8 (27%)	
	Slightly	2 (5%)	3 (9%)		2 (6%)	0	
	Not at all	12 (32%)	12 (38%)		7 (21%)	6 (20%)	
	Missing	1					
Total score		12 (0–42)	13 (0–54)	0.86	6 (0–40)	3 (0–30)	0.217

Regarding health-related quality of life, there were no significant differences between the groups at any time point when comparisons were made between baseline and follow-up at 3 and 12 months for patients who completed both measurements (Supplementary Table). At baseline, a significant difference was found for financial difficulties (*p* = 0.045) and a clinically significant difference for body image (12 points), both favouring the intervention group. Generally, health-related quality of life deteriorated from inclusion to the 3-month follow-up in both groups, with significant worsening in seven scales. The largest deterioration (> 20 points) was for trouble with coughing, and > 10 points for role functioning, nausea and vomiting, appetite loss, diarrhoea, eating, and weight loss. Six scales deteriorated > 10 points in one of the groups. Most functions recovered by the 12-month follow-up, with only one clinically significant improvement in both groups for problems with taste.

When comparing the change in scores between inclusion and the 3-month follow-up, no significant differences were found between the groups for any scales on the three questionnaires. The scores between the two groups from inclusion in the study until the 12-month follow-up were also compared and no significant differences found. Clinically significant differences were found in favour for the intervention group (the change between the two time-points differed > 10 points) for insomnia, body image, problems with reflux, and eating with others, whereas the control group improved 23 points for hair loss compared to no improvement in the study group.

## Discussion

There is increasing evidence that prehabilitation has an impact in patients undergoing major abdominal and cardiac surgery, and oesophageal surgery is not an exception [7, 31, 32]. The results of this study confirm the results of earlier trials, with positive postoperative effects on respiratory function. These findings are of great clinical importance because the most common complications in patients undergoing oesophageal surgery are pulmonary complications, which may be severe or even fatal. This study not only demonstrates beneficial effects on MIP, but also on spirometry and the number of postoperative pulmonary complications. Therefore, it is important to offer prehabilitation training interventions to patients planned for oesophageal resection.

The intervention in this study included three different activities: respiratory muscle training, strength training, and increased physical activity. The first two were structured, whereas the latter was based on the patient’s preferences and abilities. Our results indicate a more pronounced effect with the respiratory muscle training, but the impact of the other parts of the intervention are unknown. According to the logbooks, the patients performed their training according to the advice they were given and even more than instructed. It is plausible that they did so because they wanted to increase their fitness as much as possible before surgery and to be able to return to their pre-operative levels sooner. However, there were no significant differences between groups in minutes per week of physical activity or physical training. The intervention groups did not perform the tests of physical capacity or strength significantly better than the control group. The reason for this is unclear. One hypothesis is that the training was not strenuous enough to improve physical capacity. Another reason may be that also the patients in the control-group were physically active. Thus, our interpretation is that the respiratory muscle training seems to have had the most impact.

Respiratory muscle training encompasses both inspiratory muscle training (IMT) and expiratory muscle training (EMT). However, most prehabilitation studies have focused on IMT. Both IMT and EMT involve breathing with increased tidal volumes and airflow, resulting in enhanced ventilation. Deep breathing is particularly advantageous in the postoperative period, and following IMT, it can be achieved with reduced work of breathing. EMT may also offer clinical benefits, as maximal expiratory pressure (MEP) is closely associated with the ability to cough and huff effectively, which is essential for clearing airway secretions. Retention of pulmonary secretions is a common complication following oesophageal surgery and may contribute to explain the fewer pulmonary complications in the intervention group in this study. Currently, there is no consensus regarding the optimal modality of respiratory muscle training, whether IMT alone, EMT alone, or a combination of both, and further research is warranted to establish evidence-based guidelines.

In contrast to an earlier trial in which a pressure-dependent system was used, the patients in the present study performed their respiratory muscle training using a flow-dependent device [33]. The flow-dependent devices have the advantage of enabling the patient to maintain a more normal breathing rhythm, in contrast to a pressure-dependent device that produces a more jagged rhythm. With a flow-dependent device, the patients need to be instructed on its use to achieve the desired pressures, and a manometer may be connected to the device for feedback to the patient.

Even though the intervention was shown to be effective regarding pulmonary outcomes, there were no significant differences between the intervention and control groups concerning physical capacity, physical activity level, physical performance, or grip strength. Both groups appear to have been similarly active pre-operatively and continued to be postoperatively. Of course, it is possible that an extended period of training or training at a higher intensity level or frequency may have an impact on these measures. Taken together, the results suggest that respiratory IMT/EMT training before oesophageal resection surgery is of specific importance compared to physical activity in general.

The intervention did not have any impact on the quality of recovery. Recovery is multidimensional, and there are many factors that may influence the pace of postoperative improvement. Three months after discharge, the items covering physical symptoms and physical functions were not as recovered as the psychological, social, and activity items. More effort has to be invested in improving the physical aspects of a patient’s recovery. In addition, one-third of the patients rated that they were not at all recovered after 3 months and 20% rated no recovery after 12 months. Future trials, both qualitative and quantitative, are needed to explore which aspects of the recovery are involved in the low rates and how changing care can improve it.

Health-related quality of life deteriorated significantly between inclusion in the study and the 3-month follow-up in both groups. This is in line with previous descriptive studies of the impact of treatment for oesophageal cancer [34, 35]. When the health-related quality of life was compared between the two groups, no significant differences were found, but a few clinical differences were present. When focusing on the scales of special interest for this study regarding the effect of the intervention 3 months after surgery, both groups scored significantly worse for physical functioning, problems with dyspnoea, and trouble with coughing, but the latter deteriorated less in the intervention group (-21.2 vs. -31.1 points, difference = 9.9 points). We conclude that the intervention group did not benefit from the intervention in regard to health-related quality of life. One could speculate whether the impact of the disease and treatment during this period is too great, making it difficult to affect the health-related quality of life with an intervention of this kind.

This study has strengths and limitations that need to be taken into consideration. This was a randomised multicentre study conducted in five university hospitals with well-educated physiotherapists using the same kind of equipment. In addition, it was a single-blinded evaluation. However, the trial lasted 4.5 years until the required power was reached. During this period, the COVID-19 pandemic affected Sweden, leading patients to avoid any unnecessary hospital visits and decreasing follow-ups. Patients also dropped out due to changes in procedure, surgical complications, and recurrence and we did not reach the anticipated number of patients in the postoperative follow up at three months. However, the minor difference between the groups in primary outcome would not have reached the level of significance even if the number of patients were reached. The interventions used in this trial were not supervised or objectively measured; however the patients daily recorded their activities in a logbook. An addition of a pedometer or an accelerometer would have given more accurate recordings of the activity level; however, the intervention reflects conditions in clinical practice. Conducting trials in such a frail patient group undergoing extensive procedures is challenging, but these studies are essential to improving care, preventing pulmonary complications, and facilitating recovery.

In conclusion, a peri-operative training intervention including respiratory muscle training may have a positive impact on respiratory function and decrease pulmonary complications. It was shown to be safe and, therefore, can be offered to patients planned for oesophageal surgery as part of a prehabilitation programme.

## Supplementary Information

Supplementary Material 1.

## Data Availability

The dataset used during the current study is available from the corresponding author on reasonable request.

## Abbreviations

CG: Control Group
DRI: Disability Rating Index
EMT: Expiratory Muscle Training
EORTC: European Organisation of Treatment and Research of Cancer
FEV_1_: Forced Expiratory Volume during one Second
FVC: Forced Vital Capacity
IG: Intervention Group
IMT: Inspiratory Muscle Training
MEP: Maximum Expiratory Pressure
MIP: Maximum Inspiratory Pressure
PEF: Peak Expiratory Pressure
PEP: Positive Expiratory Pressure
PRP: The Postoperative Recovery Profile
6MWD: 6-minute walking distance
