# Supplementary material for: Positive effects of a perioperative training intervention in Ivor Lewis oesophageal surgery: a randomised, controlled multicentre trial
Source: BMC Surg. 2025 Dec 11;26:34. doi: 10.1186/s12893-025-03416-4 (PMC12801523; doi:10.1186/s12893-025-03416-4)
Supplement: Supplementary file 1 — Supplementary Material 1. [file 12893_2025_3416_MOESM1_ESM.doc]

Supplementary Table 1. Physical activity (PA) and physical training (PT) before and three and twelve months after oesophagostomy.

|  | | Preoperatively | | | Three months | | | Twelve months | | |
| --- | --- | --- | --- | --- | --- | --- | --- | --- | --- | --- |
|  |  | IV  n=52 | CG  n=48 | p-value | IV  n=36 | CG  n=30 | p-value | IV  n=33 | CG  n=29 | p-value |
| Physical activity, min/ week | | 308 (291) | 252 (230) | 0.315 | 263 (196) | 224 (204) | 0.840 | 322 (261) | 382 (444) | 0.245 |
| Physical training, min/week | | 63 (109) | 40 (98) | 0.140 | 38 (71) | 36 (55) | 0.940 | 59 (93) | 101 (149 | 0.079 |
| Physical activity level, n (%) | 0 | 0 | 0 | 0.649 | 0 | 0 | 0.175 | 0 | 1 (3%) | 0.602 |
|  | 1 | 0 | 2 (4%) |  | 1 (3%) | 2 (7%) |  | 1 (3%) | 2 (7%) |  |
|  | 2 | 8 (16%) | 8 (18%) |  | 4 (11%) | 3 (10%) |  | 0 | 1 (3%) |  |
|  | 3 | 14 (28%) | 11 (24%) |  | 7 (19%) | 14 (47%) |  | 8 (24%) | 3 (10%) |  |
|  | 4 | 22 (44%) | 21 (47%) |  | 22 (61%) | 8 (27%) |  | 17 (52%) | 14 (48%) |  |
|  | 5 | 3(6%) | 1 (2%) |  | 1 (3%) | 2 (7%) |  | 3 (9%) | 2 (7%) |  |
|  | 6 | 3 (6%) | 2 (4%) |  | 1 (3%) | 1 (3%) |  | 4 (12%) | 6 (21%) |  |
|  | No | 2 | 3 |  |  |  |  |  |  |  |
| PA>150 min/w | | 33 (63%) | 23 (48%) | 0.109 | 29 (81%) | 17 (57%) | 0.062 | 21 (75%) | 25 (71%) | 0.896 |
| Physical function, DRI score | | 15.8 (0;69) | 22.6 (0;52) | 0.275 | 27.1 (0;71) | 22.6 (0;52) | 0.948 | 16.1 (0;82) | 18.9 (0;66) | 0.701 |
| DRI, disability level | No | 5 | 2 | 0.255 | 1 | 1 | 0.558 | 4 | 4 | 0.948 |
|  | Some | 9 | 6 |  | 4 | 4 |  | 8 | 6 |  |
|  | Mild | 15 | 12 |  | 20 | 13 |  | 16 | 12 |  |
|  | Moderate | 10 | 1, |  | 12 | 9 |  | 5 | 6 |  |
|  | Serious | 1 | 0 |  | 4 | 4 |  | 2 | 1 |  |

Intervention group IV, Disability: No (0-1), Some (2-10), Mild (10-30), Moderate (31-65), Serious (66-100)

Supplementary Table 2. Comparison of health-related quality of life between the study and control group, for the EORTC QLQ-OG25

|  | **Inclusion** | | | **3 months** | | **Change between**  **baseline and 3 months** | | | **12 months** | | **Change between baseline and 12 months** | |  |
| --- | --- | --- | --- | --- | --- | --- | --- | --- | --- | --- | --- | --- | --- |
| **Variable** | **IG**  mean (SD) | **CG**  mean (SD) | ***p* value** | **IG**  mean (SD) | **CG**  mean (SD) | **IG**  Mean  difference (SD | **CG**  Mean  difference (SD) | ***p* value** | **IG**  mean (SD | **CG**  mean (SD) | **IG**  mean (SD | **CG**  mean (SD) | ***p* value** |
| **EORTC QLQ-OG25** | n=41 | n=35 |  | n=37 | n=32 | n=33 | n=32 |  | n=34 | n=32 | n=31 | n=30 |  |
| **Body image** | 83.8  (23.1) | 71.6 (29.7) | 0.08 | 77.5  (31.5) | 75.3  (32.2) | -7.1  (36.1) | +3.8  (30.2) | 0.27 | 76.5 (33.4) | 83.9 (29.0) | -5.4  (37.6) | +10.7  (36.3) | 0.13 |
| **Dysphagia** | 14. (24.1) | 15.2 (24.7) | 0.93 | 17.7  (18.4) | 20.5  (21.7) | -7.4 (20.9) | -3.8  (29.7) | 0.61 | 12.1 (19.8) | 6.7  (12.9) | -0.4  (31.0) | +5.2  (24.0) | 0.48 |
| **Eating** | 28.4 (28.0) | 31.6 (33.3) | 0.68 | 46.6 (26.2) | 43.5 (28.4) | -22.0  (21.2) | -11.3  (35.2) | 0.15 | 25.7 (25.7) | 22.4  (23.8) | -1.9  (30.6) | +5.1  (36.2) | 0.44 |
| **Reflux** | 17.6 (20.8) | 10.0 (17.2) | 0.12 | 18.5 (19.6) | 19.3 (24.7) | -2.0 (22.7) | -8.8  (27.1) | 0.31 | 20.6 (24.3) | 28.2 (30.9) | -6.5.  (31.5) | -18.4  (29.7) | 0.15 |
| **Odynophagia** | 18.5  (25.8) | 18.1  (25.4) | 1.00 | 17.6 (20.4) | 20.3 (23.5) | 0  (27.0) | -1.6  (24.4) | 0.89 | 17.2  (22.3) | 11.5  (21.9) | -1.1 (28.3) | +5.8 (30.3) | 0.42 |
| **Pain and discomfort** | 18.9 (27.5) | 15.2 (23.0) | 0.59 | 23.9  (22.1) | 24.0  (28.7) | -5.6  (35.3) | -5.9  (36.1) | 1.0 | 25.0 (20.6) | 27.6 (28.3) | -4.3  (32.8) | -10.1  (30.5) | 0.53 |
| **Anxiety** | 45.0 (22.9) | 45.2 (27.9) | 1.00 | 45.5 (28.0) | 48.4 (29.1) | +2.0  (28.2) | -1.0  (30.2) | 0.73 | 40.2 (31.5) | 33.9 (29.0) | +8.1  (33.9) | +11.5  (35.4) | 0.75 |
| **Eating with others** | 9.3 (18.9) | 10.8 (26.9) | 0.92 | 18.0  (24.3) | 13.5  (29.2) | -8.1 (25.0) | -3.2  (37.7) | 0.63 | 13.7 (26.1) | 8.3  (21.5) | -6.7  (28.2) | +4-9  (27.3) | 0.17 |
| **Dry mouth** | 21.6 (29.6) | 27.6 (26.6) | 0.52 | 33.3 (35.1) | 29.2 (25.0) | -12.1  (32.1) | -3.1  (34.2’) | 0.34 | 20.6 (23.2) | 25.3  (27.7) | +2.1  (29.7) | +1.2  (33.9) | 1.0 |
| **Trouble with taste** | 33.3  (38.5) | 35.2  (37.9) | 0.91 | 33.3 (32.4) | 29.2 (30.2) | -1.0 (55.6) | +5.2  (41.6) | 0.67 | 17.6  (31.0) | 12.6  (30.1) | +16.1  (45.4) | +19.5  (40.4) | 0.84 |
| **Trouble with swallowing saliva** | 8.1 (19.9) | 8.1  (22.1) | 1.00 | 0  (16.9) | 5.6  (34.0) | -7.1 (28.6) | -14.6  (28.0) | 0.51 | 6.9  (21.4) | 7.8  (16.8) | +1.1 (31.6) | -1.2  (30.7) | 0.88 |
| **Choked when swallowing** | 9.3  (15.1) | 11.1  (23.1) | 0.86 | 10.8  (17.7) | 42.7  (37.1) | -2.0  (18.5) | -8.9  (37.07) | 0.43 | 23.5  (26.6) | 12.2  (18.5) | -13.3  (20.7) | -3.6  (21.0) | 0.13 |
| **Trouble with coughing** | 25.2 (25.3) | 25.3 (26.4) | 1.00 | 44.1  (30.5) | 22.9  (28.6) | -21.2  (35.2) | -31.1  (33.8) | 0.32 | 32.4  (26.6) | 27.8  (23.3) | +8.6  (31.0) | -6.0  (31.5) | 0.86 |
| **Trouble talking** | 3.6  (10.5) | 5.1  (16.9) | 0.88 | 7.2  (16.0) | 14.6  (28.0) | -4.0  (18.1) | -7.8  (31.2) | 0.68 | 3.9  (10.9) | 11.1  (25.3) | 0  (17.2) | -6.0  (31.5) | 0.44 |
| **Weight loss** | 18.9  (24.3 | 23.2  (29.4) | 0.61 | 30.6  (28.7) | 33.3  (32.8) | -13.1  (30.0) | -10.0  (39.3) | 0.82 | 23.5  (24.0) | 30.0  (32.0) | -7.3  (35.2) | -4.8  (34.8) | 0.87 |
| **Hair loss** | 19.4  (25.9) | 25.7  (31.2) | 0.44 | 26.7  (31.7) | 16.7  (17.1) | -2.1  (25.7) | +18.7  (27.1) | 0.06 | 23.5 (32.8) | 12.1  (16.8) | 0  (29.8) | +23.3  (27.4) | 0.09 |

EORTC QLQ-OG25: The European Organization for Research and Treatment of Cancer Quality of Life Questionnaire for oesophageal-gastric cancer. A high score for body image, the better function and a high score for the symptom scales represents a greater degree of problem.

+: an improvement between baseline and a follow-up within a group, - a worsening between baseline and a follow-up within a group.

Supplementary Table 3. Comparison of health-related quality of life between the study and control group, for the EORTC QLQ-C30 and EORTC QLQ-FA12

|  | **Inclusion** | | | **3 months** | | **Change between**  **baseline and 3 months** | | | **12 months** | | **Change between baseline and 12 months** | |  |
| --- | --- | --- | --- | --- | --- | --- | --- | --- | --- | --- | --- | --- | --- |
| **Variable** | **Study**  **Group**  mean (SD) | **Control**  **Group**  mean (SD) | ***p* value** | **Study**  **Group**  mean (SD) | **Control**  **Group**  mean (SD) | **Study**  **Group**  Mean  difference (SD | **Control**  **Group**  Mean  difference (SD) | ***p* value** | **Study**  **Group**  mean (SD | **Control**  **Group**  mean (SD) | **Study**  **Group**  mean (SD | **Control**  **-Group**  mean (SD) | ***p* value** |
| **EORTC QLQ-C30** | n=41 | n=35 |  | n=37 | n=32 | n=33 | n=32 |  | n=34 | n=32 | n=31 | n=30 |  |
| **Physical functioning** | 86.7 (15.0) | 83.3 (13.0) | 0.94 | 76.7  (17.7) | 77.9  (18.6) | -10.1  (19.4) | -8.75  (17.2) | 0.80 | 82.7 (18.3) | 85.1 (16.6) | -3.8  (15.3) | -1.6  (19.5) | 0.13 |
| **Role functioning** | 75.7 (29.8) | 75.7 (29.2) | 1.00 | 63.0  (33.1) | 63.0  (37.3) | -13.1 (41.2) | -13.0  (34.3) | 1.0 | 76.5 (31.5) | 80.6 (34.5) | 0  (33.3) | +1.7  (41.8) | 0.91 |
| **Emotional functioning** | 83.3 (17.8) | 85.0 (20.3) | 0.75 | 80.9 (17.6) | 77.3 (24.0) | -2,3  (18.0) | -7.5  (16.0) | 0.24 | 81.9 (18.5) | 83.1  (20.6) | +0.8  (20.2) | -2.0  (18.7) | 0.62 |
| **Cognitive functioning** | 89.2 (17.2) | 88.2 (15.1) | 0.90 | 84.2 (21.9) | 84.9 (18.1) | -4.0 (14.5) | -3.2  (11.7) | 0.92 | 84.8 (19.8) | 88.9 (17.7) | -4.8.  (19.8) | +0.58  (19.2) | 0.35 |
| **Social functioning** | 79.7  (19.7) | 73.5  (21.4) | 0-25 | 68.9 (26.7) | 70.8 (28.1) | -9.9 (28.9) | -2.7  (24.4) | 0.38 | 83.3  (22.8) | 83.3  (22.3) | + 5.9 (24.9) | +6.3 (24.2) | 1.00 |
| **Global health status** | 66.2 (22.2) | 64.5 (20.2) | 0.77 | 59.0  (19.7) | 63.5  (16.8) | -8.1  (21.5) | -0.54  (21.7) | 0.18 | 68.6 (24.5) | 70.8 (18.4) | +3.8  (26.0) | + 4.6  (24.8) | 0.94 |
| **Fatigue** | 32.1 (27.8) | 31.1 (22.2) | 0.90 | 38.7 (19.2) | 43.4 (29.5) | -6.1  (21.5) | -12.7  (25.8) | 0.37 | 30.1 (22.3) | 34.4 (26.2) | +2.5  (31.5) | -5.2  (27.5) | 0.32 |
| **Nausea & vomiting** | 14.4 (16.7) | 12.9 (17.7) | 0.79 | 27.0  (29.0) | 24.0  (19.4) | -14.6 (36.5) | -10.9  (20.1) | 0.66 | 20.6 (28.1) | 12.8 (16.2) | -9.7  (36.5) | -0.5  (19.8) | 0.26 |
| **Pain** | 17.6 (25.4) | 14.8 (20.5) | 0.68 | 16.7 (20.4) | 24.0 (26.4) | +1.0  (33.3) | -8.85  (27.43) | 0.22 | 14.2 (21.8) | 20.0  (28.5) | +1.0  (34.7) | -3.3  (26.8) | 0.63 |
| **Dyspnea** | 29.7  (24.6) | 30.5  (21.9) | 1.00 | 43.2 (27.1) | 38.5 (26.9) | -11.1 (31.9) | -9.4  (31.9) | 0.92 | 30.4  (23.7) | 34.5  (28.8) | 0  (32.3) | -6.9  (39.2) | 0.53 |
| **Insomnia** | 27.9 (34.7) | 20.0 (29.4) | 0.36 | 31.5  (32.3) | 34.5  (28.0) | -7.1 (28.6) | -14.6  (28.0) | 0.36 | 23.5 (29.0) | 26.7 (30.8) | +5.4 (28.7) | -6.7  (37.6) | 0.20 |
| **Appetite loss** | 25.0  (30.2) | 25.7  (35.3) | 1.00 | 40.7  (38.3) | 42.7  (37.1) | -17.7  (41.5) | -16.7  (42.3) | 1.00 | 18.6  (28.7) | 13.3  (24.1) | +4.4  (33.6) | +8.9  (36.2) | 0.71 |
| **Constipation** | 16.2 (21.7) | 14.3 (30.6) | 0.88 | 13.5  (21.5) | 12.5  (22.0) | -1.0  (29.4) | +3.1  (40.4) | 0.73 | 9.8  (25.3) | 13.3  (24.1) | +5.4  (29.9) | -3.3  (32.0) | 0.34 |
| **Diarrhea** | 13.5  (22.9) | 15.7  (23.5) | 0.83 | 32.4  (26.6) | 28.1  (29.5) | -18.2  (33.4) | -11.8  (29.2) | 0.50 | 19.6  (20.3) | 22.2  (32.0) | -5.4  (28.7) | -9.2  (43.6) | 0.78 |
| **Financial difficulties** | 3.6 (10.5) | 14.7 (28.7) | 0.045 | 9.9  (22.0) | 14.6  (28.0) | -7.1  (20.0) | 0  (14.9) | 0.18 | 7.8  (16.5) | 10.0 (23.4) | -5.4  (12.5) | +1.2  (14.0) | 0.12 |
| **EORTC QLQ-FA12** |  |  |  |  |  |  |  |  |  |  |  |  |  |
| **Physical fatigue** | 34.1 (27.9) | 30.1 (21.2) | 0.53 | 38.5  (21.4) | 36.5  (27.7) | -4.3  (28.2) | -6.3  (22.3) | 0.78 | 29.4  (24.5) | 23.1  (26.0) | +4.7  (29.2) | -3.8  (22.5) | 0.92 |
| **Emotional fatigue** | 17.1  (22.9) | 15.9  (21.3) | 0.86 | 21.6  (19.6) | 20.8  (24.6) | -5.1  (23.3) | -3.2  (26.5) | 0.50 | 17.0  (21.8) | 13.0  (20.2) | +1.8  (25.8) | +4.1  (20.2) | 0.66 |
| **Cognitive fatigue** | 8.1 (18.7) | 10.5  (18.6) | 0.66 | 9.5  (18.2) | 14.6  (28.0) | -1.0  (18.6) | +1.1  (13.6) | 0.70 | 4.9  (9.65) | 10.0  (23) | +4.8  (19.8) | -1.7  (22.5 | 0.28 |

EORTC QLQ-C30: The European Organization for Research and Treatment of Cancer Quality of Life Questionnaire-Core 30. EORTC QLQ-FA12 Quality of Life Questionnaire for fatigue. A high score for global quality of life and the functional scales the better function and a high score for the symptom scales represents a greater degree of problem.

+: an improvement between baseline a follow-up within a group, - a worsening between baseline and the follow-up within a group.
